# The Interaction Between Caudate Nucleus and Regions Within the Theory of Mind Network as a Neural Basis for Social Intelligence

**DOI:** 10.3389/fncir.2021.727960

**Published:** 2021-10-14

**Authors:** Mikhail Votinov, Artem Myznikov, Maya Zheltyakova, Ruslan Masharipov, Alexander Korotkov, Denis Cherednichenko, Ute Habel, Maxim Kireev

**Affiliations:** ^1^N.P. Bechtereva Institute of Human Brain, Russian Academy of Science, Saint Petersburg, Russia; ^2^Institute of Neuroscience and Medicine, Research Centre Jülich, Jülich, Germany; ^3^Department of Psychiatry, Psychotherapy, and Psychosomatics, Medical Faculty, RWTH Aachen University, Aachen, Germany; ^4^Institute for Cognitive Studies, Saint Petersburg State University, Saint Petersburg, Russia

**Keywords:** theory of mind, precuneus, temporoparietal junction (TPJ), social cognition, social brain, caudate nucleus

## Abstract

The organization of socio-cognitive processes is a multifaceted problem for which many sophisticated concepts have been proposed. One of these concepts is social intelligence (SI), i.e., the set of abilities that allow successful interaction with other people. The theory of mind (ToM) human brain network is a good candidate for the neural substrate underlying SI since it is involved in inferring the mental states of others and ourselves and predicting or explaining others’ actions. However, the relationship of ToM to SI remains poorly explored. Our recent research revealed an association between the gray matter volume of the caudate nucleus and the degree of SI as measured by the Guilford-Sullivan test. It led us to question whether this structural peculiarity is reflected in changes to the integration of the caudate with other areas of the brain associated with socio-cognitive processes, including the ToM system. We conducted seed-based functional connectivity (FC) analysis of resting-state fMRI data for 42 subjects with the caudate as a region of interest. We found that the scores of the Guilford-Sullivan test were positively correlated with the FC between seeds in the right caudate head and two clusters located within the right superior temporal gyrus and bilateral precuneus. Both regions are known to be nodes of the ToM network. Thus, the current study demonstrates that the SI level is associated with the degree of functional integration between the ToM network and the caudate nuclei.

## Introduction

Social intelligence (SI) is defined as the set of human abilities that facilitate effective interpersonal interactions (Vernon, 1933; Shanley et al., 1971). It is closely related to the socio-cognitive processes of the theory of mind (ToM) construct, which is defined as the ability to make inferences about the mental states of others, also called mentalizing (Premack and Woodruff, 1978). Although the brain ToM system responsible for mentalizing is well studied (Molenberghs et al., 2016), the relationship between its functioning and SI remains poorly investigated. Its investigation is complicated mainly by the narrow specificity of psychometric measures of human socio-cognitive ability. Examples of such tests include the Reading the Mind in the Eyes (RMET) test (Baron-Cohen et al., 2001), and the false-belief task (Perner and Wimmer, 1985; Gopnik and Slaughter, 1991), which provide information regarding certain aspects of an individual’s mentalizing ability instead of measuring SI *per se*. Additionally, existing neuroimaging task-based studies of interpersonal interactions do not provide a cohesive view of the relationship between SI and the ToM-brain network because of limited data about neural correlates of social intelligence that would allow comparing brain organization of these entities. It can also be associated with the fact that some studies did not make a difference between SI and ToM and are considered as synonymous concepts. For example, Baron-Cohen et al. (1999) showed increased activation of the superior temporal gyrus and amygdala “when using social intelligence,” however, this study utilized the modified version of RMET.

Taking that into account, one potentially promising way of research for brain basics of social intelligence is to study the relationship between the level of SI and the characteristics of the functioning of brain networks in the resting state. To do that, one has to quantify the level of SI, which can be done by Guilford’s structure-of-intellect model (Guilford and O’Sullivan, 1976). According to this model, SI consists of 30 abilities (5 operations x 6 products) in the domain of behavioral content, of which only four can be measured using the Guilford-Sullivan test (Guilford and O’Sullivan, 1976). The subtests include missing cartoons, expression grouping, social translations, and cartoon prediction subtests. Thus, the Guilford-Sullivan test is one of the few methods available for the evaluation of socio-cognitive ability.

Recently, it was demonstrated that the gray matter volume (GMV) within the caudate nucleus was positively associated with higher SI scores as measured by the Guilford-Sullivan test (Myznikov et al., 2021). Although the caudate nucleus is known as a part of the “social brain,” there are insufficient data to demonstrate its relation to the ToM network. However, there is evidence of coactivation of the caudate and ToM-related areas during social interaction. For example, a study revealed that during live interaction with the experimenter, participants showed greater activation in ToM-associated regions such as the right temporoparietal junction (rTPJ) and anterior cingulate cortex (ACC) as well as in brain areas related to the reward system and emotion processing, including the caudate and amygdala, than they did when watching a video (Redcay et al., 2010). Additionally, direct gaze-to-gaze social interaction with a partner has been found to be associated with activity in the ventral striatum and caudate head, whereas interaction with a computer-driven agent engages areas within attention networks (Pfeiffer et al., 2014). Furthermore, the striatum has been found to be activated in settings of complex social interaction, for example, when participants consider sharing information with other individuals (Baek et al., 2017) or are performing the cooperative maze task (Krill and Platek, 2012).

In addition, there is evidence of structural connectivity and functional connectivity (FC) between the caudate nucleus and ToM-related areas. The caudate obtains projections from the prefrontal cortex, ACC, and orbitofrontal cortex (Haber, 2016). Moreover, the structural connections of the caudate nucleus link it with the precuneus and superior temporal gyrus (STG) (Selemon and Goldman-Rakic, 1985; Yeterian and Pandya, 1995), which can serve as a morphological basis for functional integration between these brain areas. This fact is in accordance with functional parcellation studies showing that a ventral part of the caudate is characterized by positive FC with the ipsilateral anterior cingulate cortex, medial and lateral prefrontal cortex, angular gyrus, and precuneus (Jung et al., 2014; Janssen et al., 2015; Kuljiš et al., 2017).

However, it is unclear how the level of socio-cognitive ability is linked to the degree of interaction between these areas. Considering the hypothetical strong relationship between the caudate and ToM system within the “social brain,” one can expect the functional interaction between them to be sensitive to and, to some degree, reflect SI. However, there is limited evidence in the literature of an impact of the SI level on the interaction between the ToM system and the caudate. Although recent voxel-based morphometry (VBM) analysis (Myznikov et al., 2021) did not reveal any correlations between the SI level and the GMV of ToM-related brain regions, it was hypothesized that the ToM network is involved in SI and can be identified via its functional interaction with the caudate nucleus as a vital region of the brain’s reward system. We performed a seed-based connectivity analysis of resting-state fMRI data and estimated correlations between SI level and functional connectivity (FC) to check this possibility. Furthermore, due to the fact that previously we observed an association between gray matter volumes in the bilateral caudate and SI scores (Myznikov et al., 2021) and that different subdivisions of the caudate nucleus are associated with distinct cognitive functions (Robinson et al., 2012; Kuljiš et al., 2017), we used seeds for functional connectivity analysis located at the head and body of caudate nucleus (CN). The functional connectivity analysis found a dorsal/ventral distinction in caudate connectivity, specifically the most dorsal caudate seed was primarily associated with DLPFC and other cognitive control regions, and the most inferior caudate seed was primarily related to limbic areas (Di Martino et al., 2008), and the similar findings were demonstrated by using diffusion tensor imaging techniques (Kotz et al., 2013). Furthermore, several functional parcellation studies of striatum demonstrated more complex functional organization of ventral and dorsal striatum and connectivity patterns of their subdivisions (Choi et al., 2012; Jung et al., 2014). The head of CN is involved in socio-cognitive processes (Kemp et al., 2013; Graff-Radford et al., 2017) and as it was revealed by meta-analytic connectivity modeling analyses that the head of the caudate corresponds closely to cognitive and emotional circuits. The body of the CN shows a strong link to action and perception related networks (Robinson et al., 2012). Therefore we assumed that functional connectivity primary between caudate head and ToM-related brain areas [including the rTPJ, precuneus, and medial prefrontal cortex (mPFC)], would be associated with the level of social intelligence according to the Guilford-Sullivan test.

## Materials and Methods

A total of 42 healthy right-handed volunteers (including 27 women) participated in the study. All participants were 24.6 ± 3.7 years old, with no history of neurological or psychological disorders and no contraindications to magnetic resonance imaging (MRI). All subjects provided written informed consent prior to their participation. All procedures were conducted in accordance with the Declaration of Helsinki and were approved by the Ethics Committee of the N.P. Bechtereva Institute of the Human Brain, Russian Academy of Sciences.

### Social Intelligence Testing

The Russian adaptation of the four-factor test of SI, developed by J. Guilford and M. Sullivan (Guilford-Sullivan test), was used to measure the level of SI (Mikhaylova, 2001). This test consists of four subtests: (1) Cartoon Predictions, (2) Expression Grouping, (3) Social Translations, and (4) Missing Cartoons (for a full description, see Myznikov et al., 2021). In the first subtest, the Cartoon Predictions, one was instructed to select one out of three cartoons, which appropriately continues the suggested situation. The second the expression grouping subtest is based on the selection of the facial expression that best fits a group of three other expressions. In the third, social translations subtest, a verbal statement between a pair of people in certain social situations is presented. Subjects are to select one out of three situations in which a suggested statement has a different meaning. In the fourth Missing Cartoons subtest, one has to complete the suggested scenario by selecting one out of four cartoons. The first subtest contained 14 trials, the second, third, and fourth subtests consisted of 15, 12, and 14 trials, respectively. Taking into account variability of activity within each subtest, the cumulative measure was used in the current study in order to get a balanced score generalized overall subtest.

### Data Acquisition

MRI was performed using a 3T Philips Achieva (Philips Medical Systems, Best, The Netherlands). Structural images were acquired using a T1-weighted pulse sequence [T1W-3D-FFE; repetition time (TR) = 2.5 ms; TE = 3.1 ms; 30° flip angle], recording 130 axial slices [field of view (FOV) = 240 × 240 mm; 256 × 256 scan matrix] of 0.94 mm thickness. Functional images were obtained using an echo planar imaging (EPI) sequence (TE = 35 ms; 90° flip angle; FOV = 208 × 208 mm; 128 × 128 scan matrix). Thirty-two continuous 3.5-mm-thick axial slices (voxel size = 3 × 3 × 3.5 mm) covering the entire cerebrum and most of the cerebellum were oriented with reference to the structural image. The images were acquired with a repetition time (TR) of 2,500 ms using 120 dynamic scans. The duration of resting-state functional MRI (rs-fMRI) scanning was 5 min. All MRI scans were inspected for image artifacts and incidental brain abnormalities. All subjects were included in the study.

### Resting-State Functional MRI Data Analysis

Preprocessing procedures were performed with the CONN functional connectivity toolbox (ver.20.c)^1^ and SPM12.^2^ Images were realigned, slice timing corrected, normalized to the standard anatomical space and smoothed with an 8 mm kernel. The Artifact Detection Tool (ART) was used to identify signal intensity spikes (global intensity z-score > 3) and fMRI volumes with excessive motion (displacement > 0.5 mm). The mean of volume being removed due to head motion was 9 ± 11. By default, CONN toolbox computed QC_timeseries [a variant of framewise displacement (FD)] that was regressed out on the first level for every subject. The mean FD for presented data was 0.1278 ± 0.059. Structural images were segmented into grey matter (GM), white matter (WM), and cerebrospinal fluid (CSF), which were then used during the denoising step. The denoising step was performed by nuisance regression. The component-based noise correction method (CompCor) strategy was used for physiological and other noise source reduction (Behzadi et al., 2007). The signals from the WM (first 5 components), CSF (first 5 components), motion parameters (6 regressors) as well as identified outliers from ART-procedure were regressed out from the functional data. After that, a band-pass filter of 0.008–0.09 Hz and linear detrending was performed.

**FIGURE 1:**
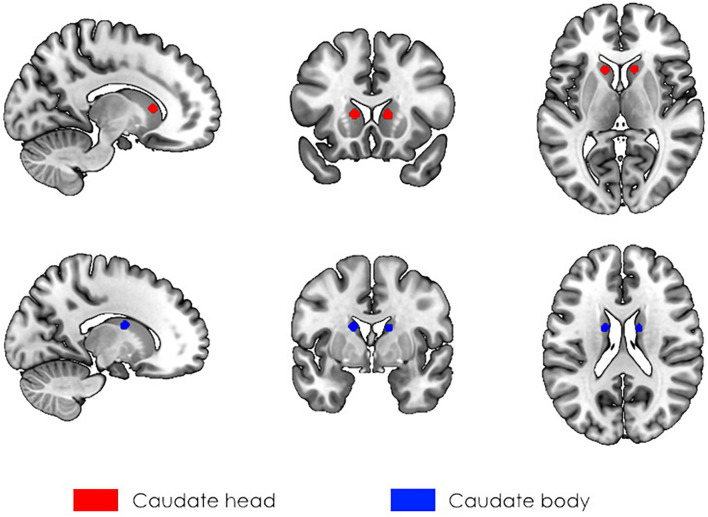
The localization of seeds used in the analysis of rs-fMRI. The 5 mm spherical masks were created for the head (red, seeds No. 1, and No. 3) and body (blue, seeds No. 2, and No. 4) of the caudate bilaterally according to Seitzman et al. (2019).

First, seed-to-voxel analysis was performed to assess the connectivity between the caudate nucleus and the rest of the brain. We selected seeds according to our previous VBM results (Myznikov et al., 2021), in which participants with high SI scores had larger values of GMV in the bilateral caudate. Thus, seeds were spheres with a 5 mm radius located in the head (right: seed No. 1, left: seed No. 3) and body (right: seed No. 2, left: seed No. 4) of the caudate with coordinates designated according to Seitzman et al. (2019) (Figure 1). The choice of seeds was made based on anatomical divisions of the caudate. We did not use the seed in the caudate tail since it is a narrowed structure in close proximity to ventricles, and the signal from that area is likely to be affected by partial volume effects (Seger, 2013). Second, at the group level analysis, the dependency of functional connectivity on the individual social intelligence level was assessed.

### Statistical Analysis

**FIGURE 2:**
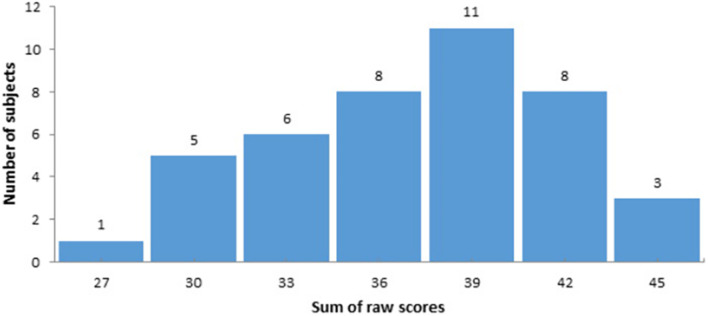
The distribution of raw scores according to the Guilford-Sullivan test of social intelligence.

Firstly, functional connectivity maps were created for each participant based on Fisher’s r-to-z transformed correlations between the mean time series in each seed and the time series of every voxel in the whole brain. To reveal a correlation between the level of social intelligence and FC between the caudate nucleus and other brain areas, the random-effect multiple regression analysis using the general linear model was used (as in implemented in the CONN toolbox). The general scores of the Guilford test were transformed into z-scores and used as a covariate of interest. The distribution of raw scores is presented in Figure 2. As well, gender and age were included as covariates of no interest. Finally, we analyzed the functional connectivity of the CN at rest. To reveal positive and negative correlations between z-transformed scores of the Guilford test and seed-to-voxel functional connectivity for the caudate nucleus ROIs the two t-contrasts were calculated for the corresponding covariate. Statistical parametric maps were created with the uncorrected *p*-value (<0.001) and a subsequent cluster-level family-wise error (FWE) correction with *p* < 0.05. The SPM results were visualized using the MRIcron toolbox.^3^

## Results

### Functional Connectivity Between Different Caudate Subdivisions and Whole-Brain Without Taking Into Account the Level of Social Intelligence

Before performing the correlation analysis between FC and the level of SI, we calculated the intrinsic functional connectivity for different caudate subdivisions within the sample. The results of this analysis are presented in the (Supplementary Figures 1–4 and Supplementary Tables 1–4). The caudate head was characterized by positive functional connectivity with the mPFC, ventrolateral PFC, and cingulate cortex and negative functional connectivity with the bilateral precuneus and superior temporal gyrus. For seeds in caudate bodies, clusters within the bilateral putamen, contralateral caudate, and thalamus were revealed. The revealed results were in accordance with previous studies (Janssen et al., 2015; Kuljiš et al., 2017).

### Results of Seed-Based Analysis and Multiple Regression With the z-Transformed Sum of Raw Scores of Social Intelligence as a Covariate of Interest

#### Seed No. 1 at Right Caudate Head

**FIGURE 3:**
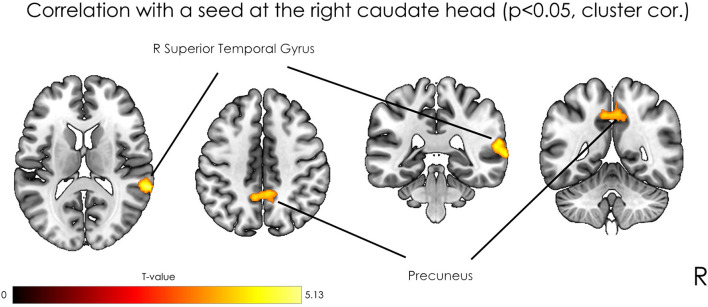
Statistical parametric maps of seed-based analysis and multiple regression with the z-transformed sum of raw scores as a covariate of interest, where the seed was located in the right caudate head, at *p* < 0.05, FWE cluster-level corrected.

**FIGURE 4:**
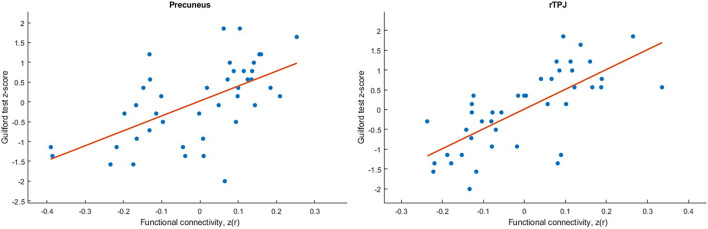
Scatterplots illustrated the association of functional connectivity between the right caudate head and both clusters in precuneus and rTPJ and Guilford test z-score.

The analysis revealed positive correlation between seed at right caudate head and clusters at right STG, bilateral precuneus, right precentral gyrus, and left postcentral gyrus. As well negative correlation was observed with a right occipital pole (Table 1). However, after applying a stricter cluster-wise FWE-corrected *p*-value (<0.05) threshold, only two clusters located within the right STG and bilateral precuneus (see Figure 3 and Table 1) survived correction. For visualization purposes, we plotted scatterplots illustrating the association of functional connectivity between the right caudate head and both clusters in precuneus and rTPJ and Guilford test z-score (see Figure 4).

**TABLE 1:** Results of the seed-based analysis and multiple regression with the z-transformed sum of raw scores included as a covariate of interest (threshold, voxel-level uncorrected *p* < 0.001, minimal cluster size (k)—30).

Region (L, left; R, right)	Cluster size (k)	T score	Cluster-level pFWE	MNI coordinates		
				x	y	Z
**Seed No. 1—right caudate head**						
**Positive correlation**						
R Supramarginal G R Superior Temporal G	473	5.25	<0.001	+64	−40	+16
Precuneus	263	3.93	<0.001	+6	−48	44
R Precentral G	43	4.07	0.72	+32	−24	+68
L Postcentral G	38	3.68	0.8	−16	−40	58
**Negative correlation**						
R Occipital Pole	32	4.00	0.88	12	−98	−14
**Seed No. 2—right caudate body**						
**Negative correlation**						
R Postcentral G	145	4.72	0.12	26	−46	58
R Cerebellum (VIII)	66	4.58	0.39	8	−74	−38
R Superior Occipital G	36	4.32	0.82	22	−70	48
L Cerebellum (Crus 1)	41	4.1	0.75	−46	−60	−22
**Seed No. 3—left caudate head**						
**Negative correlation**						
R Angular G	137	4.95	0.054	44	−58	32
**Seed No. 4—left caudate body**						
**Negative correlation**						
L Cerebellum (IV–V)	31	4.04	0.90	−24	−34	−20

#### Seed No. 2 at Right Caudate Body

The analysis revealed positive correlation between seed at right caudate body and clusters at right postcentral gyrus, right cerebellum (VIII), left cerebellum (Crus 1), and right superior occipital gyrus. As well negative correlation was observed with the right angular gyrus. However, after applying a stricter cluster-wise FWE-corrected *p*-value (<0.05) threshold, no clusters were significant (Table 1).

#### Seed No. 3 at Left Caudate Head

The negative correlation was observed with one cluster at right angular gyrus with *p* < 0.001 uncorrected threshold and it did not survive after applying cluster-wise FWE-corrected *p*-value (<0.05) threshold (Table 1).

#### Seed No. 4 at Left Caudate Body

The negative correlation was observed with one cluster at the left cerebellum (IV-V) with *p* < 0.001 uncorrected threshold and it did not survive after applying cluster-wise FWE-corrected *p*-value (<0.05) threshold (Table 1).

## Discussion

The main goal of this study was to reveal areas of the brain involved in maintaining SI by assessing FC between the caudate and other areas of the brain using rs-fMRI. We identified a positive association between the SI score and the degree of functional integration between the right caudate head and two clusters located in ToM-related regions, including the right STG and bilateral precuneus. Additionally, we revealed clusters in the precentral regions, which did not survive after correction for multiple comparisons. The results extend previous findings of a morphological relationship between the GMV of caudate nuclei and the degree of SI. The caudate nuclei are bilateral structures that consist of a head, body and tail. There is evidence of functional segregation of the caudate: a meta-analysis showed the involvement of the head of the caudate nucleus in cognition and emotion and the localization of perceptual and action-specific regions in the body of the caudate (Robinson et al., 2012). These observations can potentially explain why we observed a significant correlation between FC and Guilford scores only for the seed in the caudate head.

The precuneus and the right STG are critical nodes of the ToM network. In a meta-analysis by Molenberghs et al. (2016) these areas were reliably engaged across 144 fMRI studies. The precuneus is also known as part of the default mode network, and its activation has been observed during episodic memory retrieval, visuospatial imagery, self-processing tasks such as self-referential judgment, first- vs. third-person perspective taking and social cognition (Cavanna and Trimble, 2006). The rTPJ, which includes the superior temporal sulcus (STS) and STG, is involved in a variety of socio-cognitive processes associated with ToM. Classically, this region takes part in inferring the mental states of others as well as their goals and intentions (Saxe and Wexler, 2005). Moreover, STS activation reflects the observation of the biological movements of the eyes, mouth, hands, and body in a social context (Allison et al., 2000). Additionally, structural differences in the precuneus and TPJ are associated with different levels of socio-cognitive ability (Coutinho et al., 2013; Sato et al., 2016). A recent study showed a positive correlation between the level of self-consciousness and the GMV of the precuneus (Morita et al., 2021). This region has also been found to be associated with social-cognitive processes in the setting of deceptive behavior (Lisofsky et al., 2014; Volz et al., 2015; Kireev et al., 2017; Zheltyakova et al., 2020) as well as moral cognition (Fede and Kiehl, 2019). ToM can be fractioned into cognitive and affective domains; the cognitive domain is more likely to be included in the SI concept. Such an assumption can explain why we found clusters in the rTPJ and precuneus, which, according to fMRI studies, are attributed to cognitive ToM (Schlaffke et al., 2015; Gao et al., 2019).

A potential explanation of the presented results involves social rewards. Different social stimuli engage the neural reward system (Bhanji and Delgado, 2014), which can motivate behavior to acquire valued goals. In this context, a social reward may be characterized by the interaction of the ToM network and the reward system, where the caudate is a key node. It has been assumed that processes associated with social reward can be related to personality traits, such as sociability, which reflect an interest in social interactions (Buss, 1983). Furthermore, a recent meta-analysis showed that the anticipation of social reward was associated with activity in the striatum, insula, and left inferior frontal gyrus. Correspondingly, activation in the posterior cingulate and precuneus was found during the receipt of a social reward (Martins et al., 2021). Thus, current findings indicate a functional interaction between the ToM network and the reward system as a possible neural substrate underlying socially intelligent behavior.

Our results demonstrate that the head of the caudate nucleus could be involved in socio-cognitive processes via a functional interaction with ToM-related brain regions. This interpretation is consistent with data exhibiting the reciprocity of functional interrelationships between the reward system and ToM (Krach, 2010). Likewise, interaction with a live counterpart has been shown to be associated with activation in the ToM-associated brain regions as well as brain structures associated with the reward system, including the caudate (Alkire et al., 2018). For instance, a study found that the ball-toss game condition of high-frequency interactions between players was characterized by robust activation in the ventral striatum and the precuneus (Kawamichi et al., 2016). Additionally, there has been an attempt to formalize ToM in the framework of the reinforcement learning approach (Jara-Ettinger, 2019), where the caudate plays a potential role (Schultz, 2016).

SI is a multidimensional construct that is not exclusively limited to ToM-related socio-cognitive ability. It is a much broader conception explaining multifaceted social behavior, even though SI testing and ToM-associated tasks are quite similar. Thus, it is not surprising that SI can be characterized by the involvement of several neural systems, such as the ToM and reward systems. Such involvement potentially explains the discrepancies in our recent VBM results (Myznikov et al., 2021), where we expected to find an association between the level of SI and the GMV of ToM-related regions but did not. The present results show that although the caudate is not part of the ToM network, its interaction with ToM-associated regions differs between individuals with high SI and those with low SI. However, future investigation is needed to clarify this issue.

The presented study results have some practical implications, particularly for the understanding of socio-cognitive dysfunctions/alterations in patients with autistic spectrum disorder (ASD) (Green et al., 2015; Leekam, 2016). The involvement of the caudate as well as ToM-related regions was previously shown in the pathology of ASD. For example, reduced activation in the angular gyrus, STS region, and precuneus was demonstrated in children with ASD during the interpretation of Frith–Happé animations (Kana et al., 2015). Reward processing is also impaired in ASD and appears as diminished neural responses to social rewards in the caudate (Scott-Van Zeeland et al., 2010). Moreover, atypical connectivity patterns of the caudate have been revealed in autism (Turner et al., 2006). Since social cognition impairments are at the core of ASD, changes in FC between nodes of the reward system and the ToM network can contribute to the pathogenesis of ASD.

Finally, the limitations of the presented study should be noted. The main limitation is associated with Guilford-Sullivan social intelligence testing. There is an evidence in the literature of correlation between the level of social intelligence measured by the Guilford test and general intelligence (Shanley et al., 1971; Riggio et al., 1991). We did not control the factor of general intelligence in this study, and this can be resolved in the future studies by adding the level of general intelligence as additional covariates in multiple regression analysis of functional connectivity. Another limitation is associated with scan duration used in our study. It is wel-known fact that the long scan time increases the reproducibility and reliability of rs-fMRI data analysis (Birn et al., 2013). However, some studies showed that estimates of correlation strengths stabilize during 5–6 min, and such duration can be sufficient for FC analysis (Dijk et al., 2010). Moreover, similar studies of the Theory of Mind network utilized the same scan duration (Marchetti et al., 2015; Stoffers et al., 2015; Ling et al., 2019), which can be useful in the future for consistency between studies.

## Conclusion

These results provide new insights into the neural network structure of SI. A high level of SI was characterized by enhanced functional interaction between ToM-related brain regions and the head of the right caudate nucleus, which is a key node of the brain’s reward system. The observed integration supports the idea that the reward system is involved in socio-cognitive processes. The current findings demonstrate that such involvement could be achieved via functional interaction with the ToM network. Therefore, this study expands existing knowledge of the relationship between these two systems within the “social brain” conception.

## Data Availability Statement

The raw data supporting the conclusions of this article will be made available by the authors, without undue reservation.

## Ethics Statement

The studies involving human participants were reviewed and approved by the Ethics Committee of the N.P. Bechtereva Institute of the Human Brain, Russian Academy of Sciences. The patients/participants provided their written informed consent to participate in this study.

## Author Contributions

MV, AK, and MK conceived and designed the analysis. AM, RM, and MZ collected the fMRI data. AM and MV analyzed the rs-fMRI data. AM, MV, MZ, DC, UH, MK, and AK wrote the manuscript. All authors contributed to the article and approved the submitted version.

## Conflict of Interest

The authors declare that the research was conducted in the absence of any commercial or financial relationships that could be construed as a potential conflict of interest.

## Publisher’s Note

All claims expressed in this article are solely those of the authors and do not necessarily represent those of their affiliated organizations, or those of the publisher, the editors and the reviewers. Any product that may be evaluated in this article, or claim that may be made by its manufacturer, is not guaranteed or endorsed by the publisher.

## Footnotes

1
www.nitrc.org/projects/conn

2
www.fil.ion.ucl.ac.uk/spm/

3
https://www.nitrc.org/projects/mricron
