# Supplementary material for: The Interaction Between Caudate Nucleus and Regions Within the Theory of Mind Network as a Neural Basis for Social Intelligence
Source: Front Neural Circuits. 2021 Oct 14;15:727960. doi: 10.3389/fncir.2021.727960 (PMC8552029; doi:10.3389/fncir.2021.727960)
Supplement: Supplementary file 1 [file Data_Sheet_1.docx]

**Supplementary materials**

**Figure 1. Statistical parametric maps, demonstrating the connectivity of right caudate head, p<.001 uncorrected voxel-level, p<.05 cluster-level pFWE-corrected.**

**
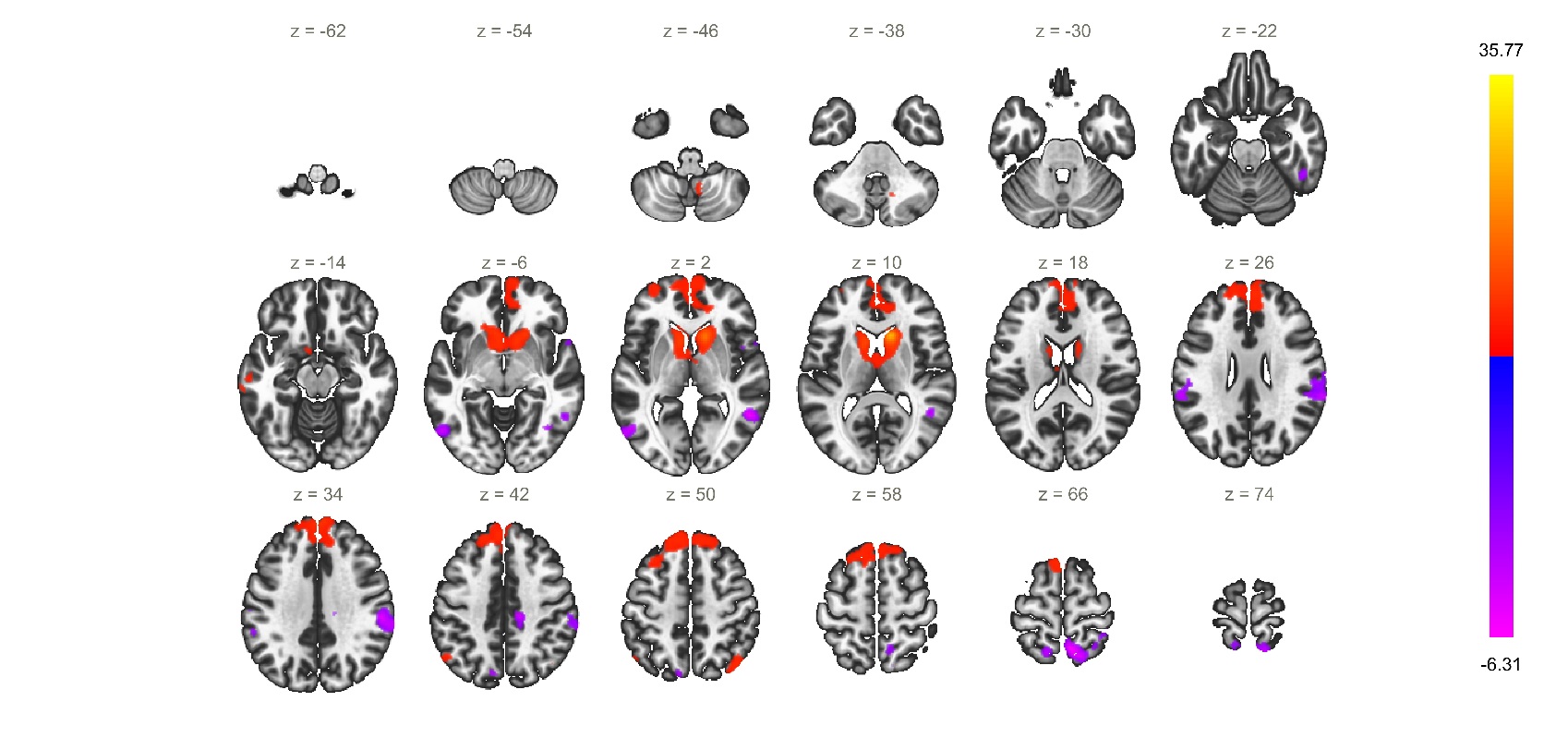
**

**Table 1. Results of functional connectivity analysis for seed in the right caudate head, p<.001 uncorrected voxel-level, p<.05 cluster level pFWE-corrected.**

| Region (L - left, R - right) | cluster size (k) | T score | Cluster  Level pFWE | MNI coordinates | | |
| --- | --- | --- | --- | --- | --- | --- |
|  |  |  |  | x | y | z |
| **One-sided positive correlation** | | | | | | |
| \| R Caudate Nucleus \| \| --- \| \| L Caudate Nucleus \| \| L Thalamus \| | 2752 | 35.77 | <0.001 | 12 | 18 | 8 |
|  |  | 9.34 |  | -10 | 12 | 10 |
|  |  | 4.39 |  | -6 | -12 | 16 |
| \| L Superior Frontal Gyrus \| \| --- \| \| L Superior Medial Gyrus \| | 6041 | 7.12 | <0.001 | -18 | 40 | 46 |
|  |  | 6.78 |  | 2 | 58 | 34 |
| R Angular Gyrus | 266 | 5.65 | <0.001 | 40 | -66 | 48 |
| L Frontal Pole | 183 | 5.13 | 0.01 | -30 | 60 | 2 |
| L Middle Temporal Gyrus | 207 | 4.30 | 0.01 | -60 | -20 | -12 |
| L Angular Gyrus | 166 | 4.13 | 0.02 | -48 | -60 | 44 |
| **One-sided negative correlation** | | | | | | |
| \| R Precuneus \| \| --- \| \| L Precuneus \| \| R Postcentral Gyrus \| | 1039 | 6.31 | <0.001 | 8 | -52 | 68 |
|  |  | 4.74 |  | -14 | -56 | 64 |
|  |  | 4.12 |  | 36 | -38 | 68 |
| R Middle Temporal Gyrus  R Fusiform Gyrus | 436 | 6.29 | <0.001 | 52 | -50 | 4 |
|  |  | 4.76 |  | 36 | -62 | -8 |
| L Inferior Temporal Gyrus | 341 | 6.03 | <0.001 | -56 | -66 | -2 |
| Posterior Cingulate | 162 | 6.01 | 0.02 | 14 | -24 | 38 |
| R Supramarginal Gyrus | 868 | 5.60 | <0.001 | 56 | -26 | 34 |
| L Superior Temporal Gyrus  L Postcentral Gyrus | 437 | 5.01 | <0.001 | -60 | -34 | 24 |
|  |  | 3.68 |  | -44 | -24 | 38 |
| R Cerebellum (Crus 1) | 181 | 4.81 | 0.01 | 46 | -44 | -26 |
| R Insula Lobe | 189 | 4.12 | 0.01 | 44 | 8 | 0 |

**Figure 2. Statistical parametric maps, demonstrating the connectivity of right caudate body, p<.001 uncorrected voxel-level, p<.05 cluster level pFWE-corrected.**

**
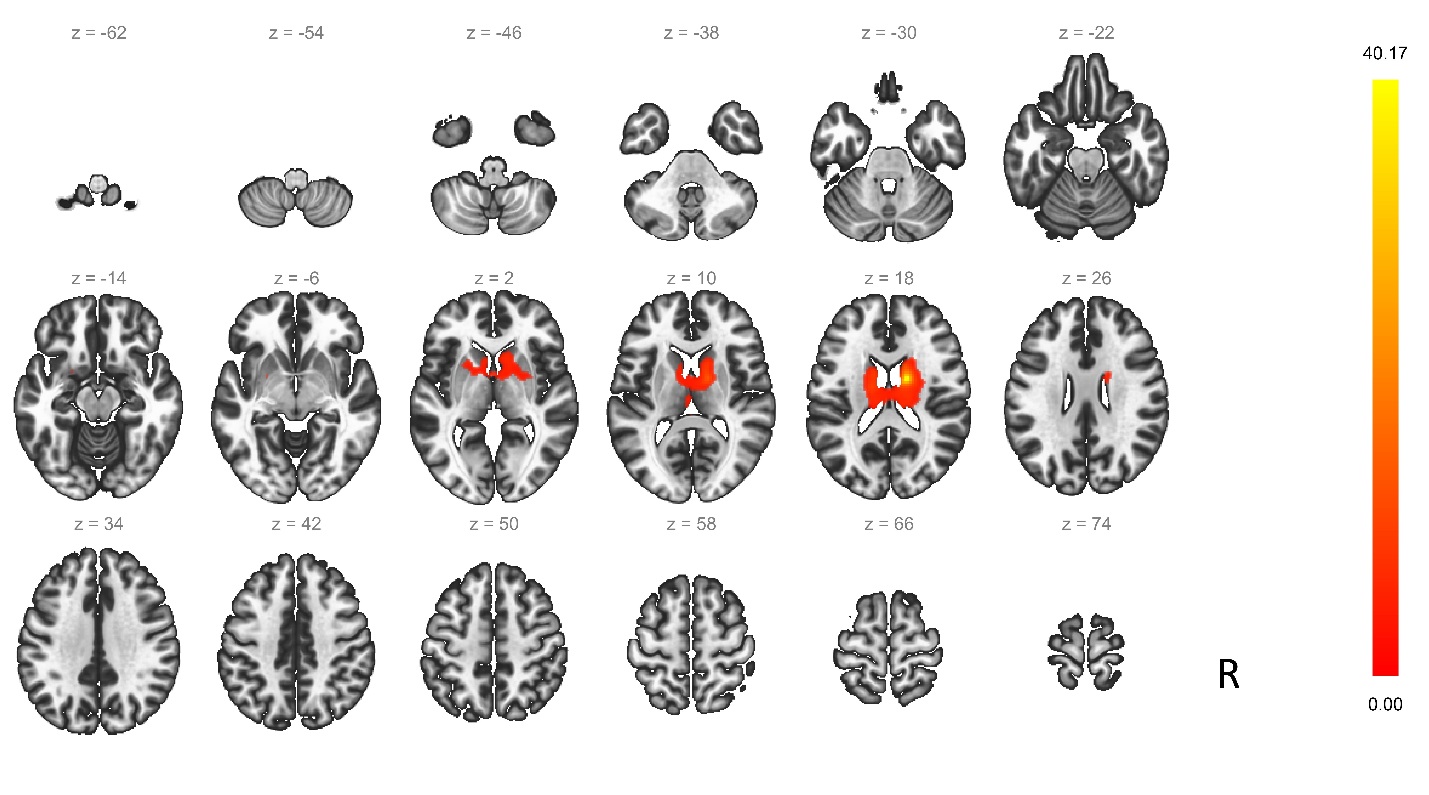
**

**Table 2. Results of functional connectivity analysis for seed in the right caudate body, p<.05 cluster level pFWE-corrected.**

| Region (L - left, R - right) | cluster size (k) | T score | Cluster  Level pFWE | MNI coordinates | | |
| --- | --- | --- | --- | --- | --- | --- |
|  |  |  |  | x | y | z |
| **One-sided positive correlation** | | | | | | |
| \| R Caudate Nucleus \| \| --- \| \| L Caudate Nucleus \| | 3173 | 40.17 | <0.001 | 14 | -2 | 18 |
|  |  | 9.72 |  | -14 | -8 | 18 |
| R IFG (p. Triangularis) | 190 | 4.36 | 0.01 | 46 | 24 | 16 |

**Figure 3. Statistical parametric maps, demonstrating the connectivity of left caudate head, p<.001 uncorrected voxel-level, p<.05 cluster level pFWE-corrected.**

**
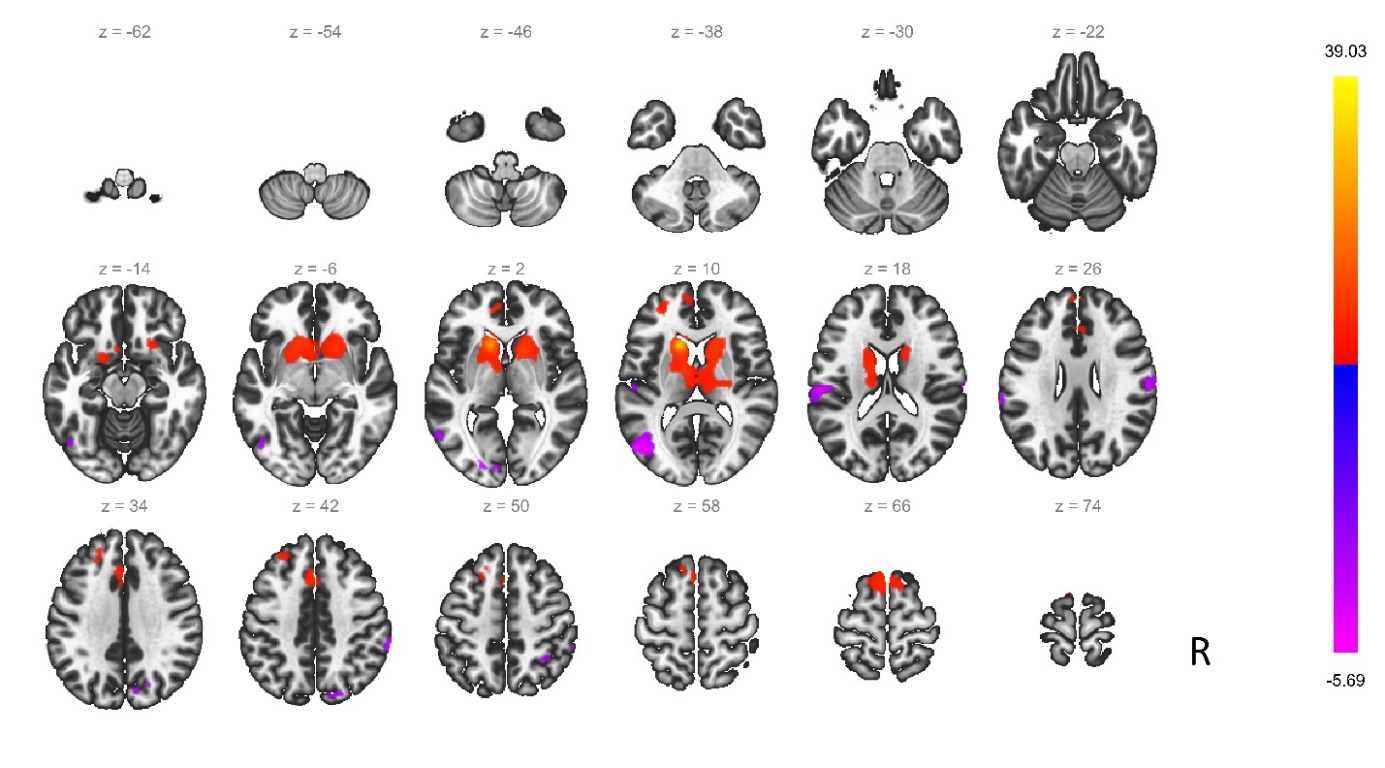
**

**Table 3. Results of functional connectivity analysis for seed in the left caudate head, p<.001 uncorrected voxel-level, p<.05 cluster level pFWE-corrected.**

| Region (L - left, R - right) | cluster size (k) | T score | Cluster  Level pFWE | MNI coordinates | | |
| --- | --- | --- | --- | --- | --- | --- |
|  |  |  |  | x | y | z |
| **One-sided positive correlation** | | | | | | |
| \| L Posterior-Medial Frontal \| \| --- \| \| L MCC \| \| L Middle Frontal Gyrus \| | 2836 | 7.1408 | <0.001 | -2 | 18 | 68 |
|  |  | 6.0763 |  | -4 | 18 | 44 |
|  |  | 5.2486 |  | -30 | 48 | 12 |
| \| L Caudate Nucleus \| \| --- \| \| R Caudate Nucleus \|   R Caudate Nucleus | 5013 | 41.1161 | <0.001 | -14 | 18 | 6 |
|  |  | 10.1379 |  | 16 | 12 | 2 |
|  |  | 8.5012 |  | -8 | -6 | 10 |
| **One-sided negative correlation** | | | | | | |
| L Middle Temporal Gyrus | 441 | 5.29 | <0.001 | -50 | -70 | 10 |
| R Inferior Parietal Lobule | 213 | 4.70 | 0.01 | 34 | -48 | 54 |
| L Postcentral Gyrus | 263 | 4.69 | <0.001 | -56 | -22 | 20 |
| L Superior Occipital Gyrus | 220 | 4.68 | 0.01 | -24 | -78 | 40 |
| R Cuneus | 333 | 4.49 | <0.001 | 14 | -80 | 42 |
| L Lingual Gyrus  R Lingual Gyrus | 522 | 4.46 | <0.001 | -20 | -86 | 0 |
|  |  | 4.36 |  | 8 | -90 | 2 |

**Figure 4. Statistical parametric maps, demonstrating the connectivity of left caudate body, p<.001 uncorrected voxel-level, p<.05 cluster level pFWE-corrected.**

**
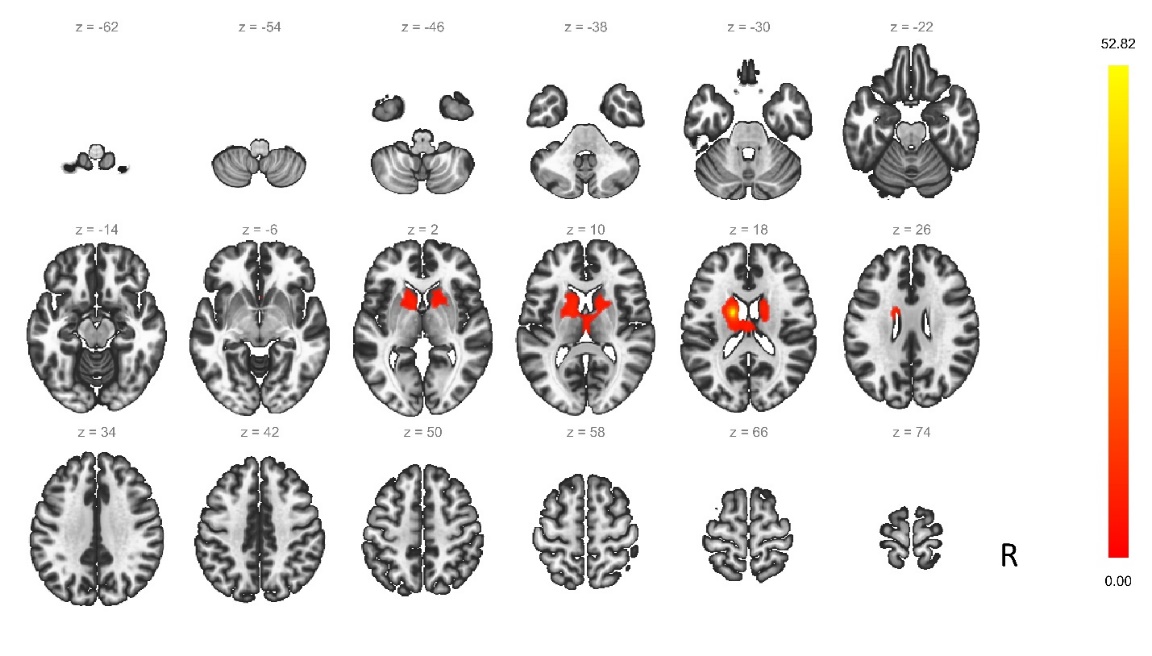
**

**Table 4. Results of functional connectivity analysis for seed in the left caudate body, p<.05 cluster level pFWE-corrected.**

| Region (L - left, R - right) | cluster size (k) | T score | Cluster  Level pFWE | MNI coordinates | | |
| --- | --- | --- | --- | --- | --- | --- |
|  |  |  |  | x | y | z |
| **One-sided positive correlation** | | | | | | |
| L Caudate Nucleus  R Caudate Nucleus  L Caudate Nucleus | 2838 | 52.8166 | <0.001 | -16 | -2 | 18 |
|  |  | 9.3944 |  | 14 | 6 | 14 |
|  |  | 7.6699 |  | -10 | 10 | 0 |
